# Inequalities in Advice Provided by Public Health Workers to Women during Antenatal Sessions in Rural India

**DOI:** 10.1371/journal.pone.0044931

**Published:** 2012-09-17

**Authors:** Abhishek Singh, Saseendran Pallikadavath, Faujdar Ram, Reuben Ogollah

**Affiliations:** 1 Department of Public Health & Mortality Studies, International Institute for Population Sciences, Mumbai, India; 2 Global Health & Social Care Unit, School of Health Sciences and Social Work, University of Portsmouth, Portsmouth, United Kingdom; Kenya Medical Research Institute - Wellcome Trust Research Programme, Kenya

## Abstract

**Objectives:**

Studies have widely documented the socioeconomic inequalities in maternal and child health related outcomes in developing countries including India. However, there is limited research on the inequalities in advice provided by public health workers on maternal and child health during antenatal visits. This paper investigates the inequalities in advice provided by public health workers to women during antenatal visits in rural India.

**Methods and Findings:**

The District Level Household Survey (2007–08) was used to compute rich-poor ratios and concentration indices. Binary logistic regressions were used to investigate inequalities in advice provided by public health workers. The dependent variables comprised the advice provided on seven essential components of maternal and child health care. A significant proportion of pregnant women who attended at least four ANC sessions were not advised on these components during their antenatal sessions. Only 51%–72% of the pregnant women were advised on at least one of the components. Moreover, socioeconomic inequalities in providing advice were significant and the provision of advice concentrated disproportionately among the rich. Inequalities were highest in the case of advice on family planning methods. Advice on breastfeeding was least unequal. Public health workers working in lower level health facilities were significantly less likely than their counterparts in the higher level health facilities to provide specific advice.

**Conclusion:**

A significant proportion of women were not advised on recommended components of maternal and child health in rural India. Moreover, there were enormous socioeconomic inequalities. The findings of this study raise questions about the capacity of the public health care system in providing equitable services in India. The Government of India must focus on training and capacity building of the public health workers in communication skills so that they can deliver appropriate and recommended advice to all clients, irrespective of their socioeconomic status.

## Introduction

Prenatal care, skilled birth attendance and postnatal care strategies have been recommended for several years [1]–[3] and are important interventions for improving maternal and child health in general and in developing countries in particular [1], [4]–[8]. According to the WHO guidelines every woman must get care during pregnancy, childbirth and postpartum period. Recognising the importance of these interventions, the Government of India has formulated a number of strategies and interventions for improving maternal and child health in the country. The National Population Policy 2000, the National Health Policy 2002, the Reproductive and Child Health Program (Phase I – 1997–2004, Phase II – 2005–2010), and the National Rural Health Mission (2005–2012), that provide antenatal (ANC), natal and postnatal care (PNC), are some important policies and interventions [9]–[12]. These policies and interventions are designed to improve the health of the general masses, especially maternal and child health among the poor and the socio-economically marginalized classes of the Indian society. The Government of India has also launched a conditional cash transfer scheme – more commonly known as *Janani Suraksha Yojana* (JSY) – under the National Rural Health Mission to promote births in the public health care system in the country. Further, maternal and child health services are provided free of charge in the public health care system. Another important step taken by the Government of India to improve accessibility of health services is to make certain primary health centres in the rural areas functional 24 hours a day.

Despite all these efforts, the utilisation of care during pregnancy, at childbirth and during the postpartum period has been limited in India. Recent data from the National Family Health Survey 2005–06 suggests that only 37% of pregnant women made 4+ antenatal visits for their most recent pregnancy, 39% delivered their recent babies in a health facility, and only 42% of women reported receiving postnatal check-up after their most recent birth [13]. Moreover, the trends in utilisation of care during pregnancy, childbirth and postpartum period over the last two decades do not reflect much improvement [13]. Not only is the utilisation of recommended care low, but there are also pronounced socioeconomic inequalities in antenatal, natal and postpartum care [14]–[16], with the rich utilising the care more than the poor.

Advice and counselling are important components of the antenatal care package and key to improving health behaviour and care seeking during pregnancy, labour and delivery and in the post-partum period [17]. The information provided during the antenatal sessions enables women and their family members to adopt health promoting behaviour and to identify and act on medical emergencies that may arise during pregnancy, delivery and post-partum periods [18]–[19]. The information provided during antenatal sessions also enables women to take proper care of their newborn. Advice on appropriate family planning methods ensures spacing between the children and preventing unwanted births. Although there is sufficient evidence to show the effectiveness of advice and counselling during antenatal sessions, there is little emphasis on advice and counselling during antenatal visits in the developing country settings [20]–[22]. Such little emphasis advice and counselling during antenatal sessions contributes to the discrepant pattern of high antenatal care but low skilled attendance at birth in Sub-Saharan African country settings [20].

There are only a few studies that have examined inequalities in advice provided during the antenatal sessions. Recent studies in rural Tanzania reported that most recommended topics critical to improving maternal and newborn survival were rarely covered during the antenatal sessions [20]–[22]. A few Indian studies have also highlighted the preference of health workers towards certain services during the antenatal sessions. A prospective study in rural Karnataka revealed that health workers were more likely to recommend iron folic acid supplements (IFA) and tetanus toxoid injections during antenatal sessions, than give advice [23]. Another study revealed that antenatal counselling on breasfeeding was inadequate [24]. Singh et al., (2011) in a recent study showed that a part of the non-use and inequalities in maternal health care can be explained by the inherent drawbacks in health systems, which treats clients according to their socioeconomic position [16]. Surprisingly, none of these studies explicitly examined the inequalities in advice provided by health workers to women during antenatal sessions in India.

The Government of India guidelines recommend that every pregnant woman have at least three antenatal visits and that the first visit is preferably in the first trimester. During the antenatal visits, pregnant women must undergo physical examination (weight and height measurement, blood pressure measurement, etc.), interventions (such as iron folic acid supplementation and tetanus toxoid vaccinations), laboratory investigations, counselling (on newborn care, institutional delivery, contraception, etc.), etc [25]. It is important to note that antenatal care is provided free of charge in public health care facilities.

Our study is, therefore, an attempt to investigate the inequalities in advice provided by the public health workers to women during antenatal sessions in rural India using a recent and representative large-scale dataset that was particularly designed to monitor the performance of the Reproductive and Child Health (RCH) program in India. In particular, this paper examines the inequalities in advice according to the wealth status of the clients. The paper investigates the inequalities in advice separately for higher level and lower level health facilities. We restrict our analysis to rural areas as the rural population depends heavily on public health facilities for maternal and child health care services.

## Data and Methods

### Ethics statement

The study is based on a secondary data set with no identifiable information on the survey participants. This dataset is available in the public domain for research use and hence no formal approval from the institutional review board is required. So, no ethics statement is required for this work.

### Data

We used data from the ‘District Level Household Survey’ round three (DLHS-3) conducted in 2007–08 in 601 districts from 34 states and union territories of India. The DLHS-3 was designed to provide estimates on maternal and child health, family planning and other reproductive health indicators at the district level [26]. The data were collected using a multi-stage stratified systematic sampling design, which resulted in national and state-representative samples after applying appropriate sampling weights to control for complex survey design [26]. The main instrument for collection of data in DLHS-3 was a set of structured questionnaires. In all 643,944 ever married women aged 15–49 years and 166,260 unmarried women aged 15–24 years were interviewed during the survey. The household and ever married woman response rates were 94 percent and 89 percent respectively. There were only small variations in the household and eligible informant response rates across different states of the country. The questions on antenatal care were asked to only those women who had live/still births in the three years preceding the DLHS-3 survey and were restricted to the most recent birth.

### Outcome variables

Dependent variables used in the analysis are the advice provided on seven essential components of maternal and child health care during antenatal sessions. These components include 1) breastfeeding, 2) keeping the baby warm, 3) spacing methods of family planning, 4) limiting methods of family planning, 5) nutrition, 6) institutional delivery, and 7) visiting a health facility in case of pregnancy complications. Advice and counselling during antenatal sessions play a critical role in preventing morbidity and mortality among the mothers and the newborn by enabling women and their family members to adopt health promoting behaviour and to identify and act on medical emergencies that may arise during pregnancy, delivery and post-partum periods [18]–[19]. We included only selected advice in the analysis since every pregnant woman is expected to have been given these advice during the antenatal sessions. To account for the willingness and co-operation on the part of the respondent, we included only those women in the analysis who have had at least four antenatal visits. We also restricted our analysis to only those women who reported visiting a public health facility for availing themselves of antenatal care.

### Exposure variables

The main explanatory variable of interest in the analysis is a measure of wealth – computed in order to investigate inequalities in access by poverty status. In the absence of direct data on income or expenditure in many surveys, a wealth index based on the ownership of household assets is widely used as a proxy for assessing the economic status of the households [27]–[33]. Besides wealth, other socio-demographic variables also have a significant impact on the utilisation of maternal and child health care. Accordingly, we included woman's education, caste, religion, woman's age, region of residence, and birth order in the analysis. We also created an indicator variable to measure the level of health facility (lower level health facility; higher level health facility). Health sub-centers (HSCs) and primary health centers (PHCs) were clubbed into lower level health facilities. The higher level health facilities included community health centers (CHCs), rural hospitals (RHs), first referral units (FRUs) and hospitals.

### Methods

To assess the extent of inequalities in access to advice, we estimated rich-poor ratios (poorest wealth quintile/richest wealth quintile), as well as concentration indices (CI). The CI measures the relationship between the accumulated proportions of individuals ranked by their socioeconomic status against the cumulative proportion of utilisation of healthcare [32], [34]–[36]. The values of the CI range from −1 to 1. A value equal to 1 or −1 indicates that only the richest or the poorest individuals use the healthcare facilities respectively. The CIs were standardised for various socioeconomic and demographic characteristics. Appropriate sampling weights were used for generating the bivariate results presented in Tables 1 and 2. Binary logistic regression models were estimated to assess the adjusted effect of wealth quintile on the likelihood of receiving advice after adjusting for relevant socioeconomic and demographic characteristics. *Wald* test was used in the binary logistic regression models to examine whether or not wealth status was a predictor of receiving advice during the antenatal sessions.

**Table 1:** Advice received during antenatal sessions held in public facilities (%), India, 2007–08.

Advice received on	Total N = 32,316	Rural N = 23,219	Level of facility (rural areas only)	
			Higher level N = 11,438	Lower level N = 11,781
Breastfeeding	74.6	72.5	75.2	69.9
Keeping baby warm	64.9	62.3	65.5	59.4
Spacing methods of family planning	55.2	52.8	55.1	50.5
Limiting methods of family planning	52.8	50.8	52.5	49.3
Nutrition	69.1	67.2	68.3	66.0
Institutional delivery	61.0	59.3	60.3	58.4
Going to a health facility in case of pregnancy complication	61.2	59.7	61.3	58.2

**Table 2:** Rich-poor ratio in provision of advice during antenatal sessions held in public facilities, India, 2007–08.

Advice received on	Total	Rural	Level of facility (rural areas only)	
			Higher level	Lower level
Breastfeeding	1.21	1.21	1.19	1.20
Keeping baby warm	1.29	1.28	1.16	1.32
Spacing methods of fp	1.36	1.36	1.23	1.39
Limiting methods of fp	1.21	1.24	1.17	1.25
Nutrition	1.21	1.21	1.20	1.20
Institutional delivery	1.23	1.26	1.23	1.27
Going to a health facility in case of pregnancy complication	1.19	1.21	1.06	1.29

Note: A ratio more than one indicates that the rich are more likely than the poor to receive the specified advice.

## Results

The advice given to pregnant women on the seven components during antenatal sessions in India was far from universal and varied considerably across the type of advice (Table 1). The advice on breastfeeding (75%) was the most common followed by advice on nutrition (69%). About 65% of pregnant women were advised on keeping the baby warm and about 61% of the women were advised on institutional delivery and visiting a health facility for pregnancy complications. Pregnant women were advised least on family planning methods for limiting (only 53% were advised on limiting methods). Rural women were only slightly less likely to receive the recommended advice during antenatal visits compared to their all-India counterparts.

The provision of advice on the seven components also varied by the level of public health facility; the chances of providing such advice being higher in higher level facilities compared to lower level facilities. For example, 75% of pregnant women who availed themselves of antenatal care in the higher level facilities were advised on breastfeeding compared to only 70% among those who availed of antenatal care in the lower level facilities. Likewise, 65% of women availing of antenatal care in the higher level facilities were given advice on keeping the baby warm compared to only 59% among those who availed themselves of antenatal care in the lower level facilities.

The rich-poor ratios suggest enormous socioeconomic inequalities in the provision of the aforementioned advices. The rich-poor ratios were consistently above one, thus indicating that the rich were more likely than the poor to receive advice (Table 2). Moreover, the rich-poor ratios were higher in the lower level public facilities compared to the higher level public facilities, indicating that inequalities were more pronounced among women who availed themselves of antenatal care in the lower level facilities. The findings based on standardised concentration indices also support the findings based on rich-poor ratios. The standardised concentration indices clearly suggest significant socioeconomic inequalities in the provision of advice, the rich being significantly more likely than the poor to receive advice even after adjusting for a number of socioeconomic and demographic characteristics (Table 3).

**Table 3:** Concentration indices for provision of advice during antenatal sessions held in public facilities, rural India, 2007–08.

Advice received on	Rural	Level of facility (rural areas only)	
		Higher level	Lower level
Breastfeeding	0.017***	0.012***	0.019***
Keeping baby warm	0.031***	0.017***	0.042***
Spacing methods of fp	0.039***	0.026***	0.050***
Limiting methods of fp	0.037***	0.036***	0.035***
Nutrition	0.025***	0.029***	0.021***
Institutional delivery	0.028***	0.026***	0.032***
Going to a health facility in case of pregnancy complication	0.019***	0.011*	0.023***

*** ***p<0.001,***

** ***p<0.01 and,***

* ***p<0.05.***

The binary logistic regression results for providing advice on the seven components during antenatal sessions are shown in Table 4. Results adjusted for relevant socioeconomic and demographic characteristics suggest that the rich were significantly more likely than the poor to receive advice during antenatal sessions. Rich women were 1.41 and 1.52 times as likely as poor women to receive advice on institutional delivery and nutrition respectively. Likewise, rich women were 1.36 and 1.38 times more likely than poor women to receive advice on breastfeeding and keeping the baby warm respectively. The other components showed a similar trend. *Wald* test was significant in all the models indicating that wealth status was indeed a predictor of receiving the seven sets of advice. Findings further suggest that women availing of antenatal care in the higher level facilities were significantly more likely to receive advice on breastfeeding, keeping the baby warm, spacing and limiting methods of family planning, and nutrition than women availing of antenatal care in the lower level facilities.

**Table 4:** Odds ratios for provision of advice during antenatal sessions held in public facilities, rural India, 2007–08.

Covariate & category	Breastfeeding	Keeping baby warm	Spacing method of family planning	Limiting method of family planning	Nutrition	Institutional delivery	Going to a health facility in case of pregnancy complications
**Wealth quintile** ‡							
Poorest (R)							
Poorer	1.06	1.03	1.13*	1.03	1.12*	1.05	1.12*
Middle	1.14*	1.17**	1.29***	1.17**	1.32***	1.19***	1.18***
Richer	1.19**	1.30***	1.33***	1.26***	1.42***	1.27***	1.23***
Richest	1.36***	1.38***	1.44***	1.38***	1.52***	1.41***	1.32***
**Level of Facility**							
Higher level (R)							
Lower level	0.92**	0.89***	0.89***	0.92**	1.06*	1.06	0.95

***Note:***

*** ***p<0.001,***

** ***p<0.01,***

* ***p<0.05,***

‡ **wealth** ***quintile was significant in ‘Wald Test’, (R) indicates the reference categories.***

***The results were adjusted for caste, religion, women's education, women's age at the birth of the index child, birth order and region of residence.***

The binary logistic regression results separately for higher level and lower level facilities are presented in Tables 5 and 6 respectively. Among women who availed themselves of antenatal care in a higher level facility, the rich were more likely than the poor to receive advice on six out of the seven components (the rich were as likely as the poor to receive advice on visiting a health facility for pregnancy complications). However, *Wald* test results suggest that wealth status was a predictor of receiving advice in only five out of seven components (the rich were as likely as the poor to receive advice on keeping the baby warm and on visiting a health facility for pregnancy complications). In contrast, among those women who availed themselves of antenatal care facilities, the rich were 1.3 to 1.5 times more likely than the poor to receive advice on all the seven components in the lower level facilities (Table 6). Indeed, the effect of wealth status was much more pronounced in the lower level facilities compared to the higher level facilities.

**Table 5:** Odds ratios for provision of advice during antenatal sessions held in higher level public facilities, rural India, 2007–08.

Covariate & category	Breastfeeding	Keeping baby warm	Spacing method of family planning	Limiting method of family planning	Nutrition	Institutional delivery	Going to a health facility in case of pregnancy complications
**Wealth quintile** ‡							
Poorest (R)							
Poorer	1.28**	1.13	1.19*	1.08	1.15	1.13	1.07
Middle	1.19*	1.04	1.09	1.09	1.30**	1.15	1.00
Richer	1.29**	1.19*	1.20*	1.26**	1.49***	1.27**	1.06
Richest	1.45***	1.28*	1.34**	1.39***	1.67***	1.44***	1.20

***Note:***

*** ***p<0.001,***

** ***p<0.01,***

* ***p<0.05,***

‡ **wealth** ***quintile was significant in ‘Wald Test’ for all except the second and the last variables, (R) indicates the reference categories.***

***The results were adjusted for caste, religion, women's education, women's age at the birth of the index child, birth order and region of residence.***

**Table 6:** Odds ratios for provision of advice during antenatal sessions held in lower level public facilities, rural India, 2007–08.

Covariate & category	Breastfeeding	Keeping baby warm	Spacing method of family planning	Limiting method of family planning	Nutrition	Institutional delivery	Going to a health facility in case of pregnancy complications
**Wealth quintile** ‡							
Poorest (R)							
Poorer	0.96	0.96	1.07	0.99	1.12	1.00	1.14*
Middle	1.15*	1.26***	1.47***	1.26***	1.38***	1.24***	1.27***
Richer	1.16*	1.39***	1.45***	1.26***	1.39***	1.30***	1.33***
Richest	1.34**	1.47***	1.48***	1.37***	1.36**	1.37***	1.33**

***Note:***

*** ***p<0.001,***

** ***p<0.01,***

* ***p<0.05,***

‡ **wealth** ***quintile was significant in ‘Wald Test’, (R) indicates the reference categories.***

***The results were adjusted for caste, religion, women's education, women's age at the birth of the index child, birth order and region of residence.***

## Discussion

According to the 2011 Indian census, around 69% of the Indian population resides in rural areas [37], and depends heavily on the public health infrastructure for their health care needs in general, and for maternal and child health services in particular. It is important to mention here that the maternal and child health services are offered free of charge to the clients in public health facilities. Contrary to our expectations, our findings suggest that the advice provided to pregnant women on the seven components during antenatal sessions in India was far from universal and varied considerably across the type of advice. Not only was the provision of advice limited, but there were also significant socioeconomic inequalities. Our study clearly shows that the rich were more likely than the poor to receive advice vital to improving maternal and child health in a low-resource setting like rural India from public health workers.

Earlier studies have clearly highlighted the important role that access and cost factors play in determining the utilisation of maternal and child health care services in India. These factors also contribute to inequalities in maternal and child health outcomes [15], [38]–[42]. Only a few studies have examined the role of health workers in perpetuating socioeconomic inequalities in maternal and child health. Our study provides evidence that the health workers proffer advice to their clients based on their own perceptions of the clients' needs. Socioeconomic status of clients was an important factor that determined the advice to be given to clients. This is consistent with the findings of various studies conducted in similar socioeconomic settings [16]–[17], [43]–[47].

That socio-economic inequalities exist in almost every sphere of activity in Indian society is well recognized. These inequalities also exist in the provision of health services where public health workers play a role in perpetuating these inequalities and are selective in providing treatment and advice. They tend to be biased in favour of the richer sections of the society. Though these findings are based on a small sample and cover only a few domains of maternal and child health, the situation might hold good in general for a large section of the Indian society, and for other critical components of health. Our findings are strongly supported by the findings of Singh et al., (2011) which also showed significant socioeconomic inequalities in the utilisation of postnatal care among facility births [16]. Socioeconomic inequalities in the utilisation of postnatal care among facility births cannot be explained simply by access and cost factors. These findings have to do more with the capacity of the public health care system in providing equitable care to its clients. Malhotra and Do (2012) also showed that the socio-economic disparities exist in health system responsiveness in India, irrespective of the type of health facility used [48].

A few studies from the developed and developing world have shown that inequity is built in the health systems [49], that public spending favours mostly the better-off rather than the poor [50]–[51], and that the delivery of health care services remains extremely unequal [52]–[53]. Our findings are consistent with the findings of these studies and bring to the forefront the socioeconomic disparities in the delivery of maternal and child health care services in the public health system in India. Recognizing the in-built inequity in the health systems of various developed and developing countries, *The World Health Report 2005* has particularly emphasized on the critical issues of exclusion and equity [1], [54]. Our findings along with others call for health system reforms to address the inequalities in the delivery of health services.

This study has some limitations. The information in DLHS-3 on advice during antenatal sessions was obtained from women and thus might be affected by recall bias. However, the chances of such bias are limited because we restricted our analysis to only those women who had four or more antenatal visits. By doing this, we included only those women in our analysis who were motivated enough to utilise the antenatal care services and thus recall the information provided to them. The analysis was also restricted only to the most recent birth that took place in the three years preceding DLHS-3. The survey format has been regularly used in the various rounds of DLHS and the findings are widely used to monitor the performance of policies and programs in India. Survey teams receive formal training and quality control measures are in place. Moreover, a study conducted in Indonesia, India and China has shown that while systematic reporting heterogeneity was significant, it was relatively small in magnitude and did not have a substantial effect on measured socio-economic inequality in health [55].

Our findings have important policy implications. The Government of India has taken a number of steps for improving maternal and child health in India. Promoting antenatal care, delivery care and postnatal care are some of the interventions. Studies have shown that there is a preference among the health workers for medical procedures during antenatal visits and advice and counselling are limited [15], [23]. This could be one of the reasons why there are significant differences between coverage of antenatal care and coverage of facility based delivery and postnatal care. Recent data suggests that about 77% of ever-married women in India utilised antenatal care services for their most recent births. This compares with only 39% of the births taking place in health facilities. The uptake of postnatal care was even more limited – only 37% received a health check-up within the critical first two days after delivery [13]. The policy makers and program managers must therefore ensure that the health workers provide specific advice and couselling to pregnant women during antenatal visits to increase the coverage of delivery and postnatal care.

In addition, the Government of India is taking a number of initiatives to improve public health care infrastructure to provide quality care to the rural and socioeconomically deprived population under its most ambitious program, the National Rural Health Mission (NRHM) 2005–2012. The Government of India has also formulated Indian Public Health Standards (IPHS) and is trying to bring health care infrastructure especially in rural areas up to IPHS. A number of other architectural corrections have also been proposed to deliver quality care to the rural population. The Government of India is planning to launch the second phase of NRHM. So, it is high time that the Government of India focused more on training and capacity building of the health personnel so that they can deliver appropriate and recommended care to the clients. Special sessions must be organised to sensitize health workers towards the special needs of the socially and deprived classes of the population, who need more care and attention. The health workers must also be informed about the rising inequalities in maternal and child health and its negative consequences. These strategies are particularly needed for public health workers deputed to the lower level health facilities, as our findings clearly reveal higher inequalities in advice among women who availed of antenatal care from lower level health facilities compared to those who availed of antenatal care from higher level health facilities. Policy-makers and program managers should think seriously about improving the managerial skills of the health workers, so that they treat their clients equally irrespective of their socioeconomic status. If immediate attention is not given to these findings, there is every chance that the public health care system will improve in terms of infrastructure, but will fail in delivering the required services to those who need them the most.
